# Heavy Metal Pollution and Health-Ecological Risk Assessment in Agricultural Soils: A Case Study from the Yellow River Bend Industrial Parks

**DOI:** 10.3390/toxics13100834

**Published:** 2025-09-30

**Authors:** Zang Liu, Li Mo, Jiahui Liang, Huading Shi, Jingjing Yao, Xiaoxiu Lun

**Affiliations:** 1 Beijing Forestry University, Beijing 100083, China; liuzang121@bjfu.edu.cn (Z.L.); lunxiaoxiu@bjfu.edu.cn (X.L.); 2 Technical Centre for Soil, Agriculture and Rural Ecology and Environment, Ministry of Ecology and Environment, Beijing 100012, China; liangjiahui@tcare-mee.cn (J.L.); shihuading@tcare-mee.cn (H.S.); 3 Institute of Resources and Environment, Beijing Academy of Science and Technology, Beijing 100095, China; yaojing1989_lucky@163.com

**Keywords:** heavy metals, source apportionment, PCA and PMF model, human health

## Abstract

Agricultural soils near industrial parks in the Yellow River bend region face severe heavy metal pollution, posing a significant to human health. This study integrated field sampling with laboratory analysis and applied geostatistical analysis, positive matrix factorization (PMF) modeling, and health risk assessment models to systematically investigate the pollution levels, spatial distribution, sources, and ecological health risks of heavy metals in the area. The main findings are as follows: (1) The average concentrations of the eight heavy metals (Hg, Cr, Cu, Pb, Zn, As, Cd, and Ni) in the study area were 0.04, 48.3, 54.3, 45.7, 70.0, 22.9, 0.4, and 35.7 mg·kg^−1^, respectively. The concentrations exceeded local background values by factors ranging from 1.32 to 11.2. Exceedances of soil screening and control values were particularly pronounced for Cd and As. Based on the geoaccumulation index, over 75% of the sampling sites for Cr, Pb, Zn, and Cd were classified as moderately to heavily polluted. Potential ecological risk assessment highlighted Cd as the significant ecological risk factor, indicating considerable heavy metal pollution in the region. (2) Kriging interpolation demonstrated elevated concentrations in the western (mid-upper) and eastern (mid-lower) subregions. Pearson correlation analysis suggested common sources for Cu-Pb-As-Cd and Cr-Zn-Ni. (3) PMF source apportionment identified four primary sources: traffic emissions (38.19%), natural and agricultural mixed sources (34.55%), metal smelting (17.61%), and atmospheric deposition (10.10%). (4) Health risk assessment indicated that the non-carcinogenic risk for both adults and children was within acceptable limits (adults: 0.065; children: 0.12). Carcinogenic risks were also acceptable (adults: 5.67 × 10^−5^; children: 6.70 × 10^−5^). In conclusion, priority should be given to the control of traffic emissions and agriculturally derived sources in the management of soil heavy metal contamination in this region, while the considerable contribution of smelting activities warrants heightened attention. This study provides a scientific basis for the prevention, control, and targeted remediation of regional soil heavy metal pollution.

## 1. Introduction

Rapid industrialization and urbanization in China have led to increased pollutant emissions and a decline in environmental quality [1]. Of particular concern is soil pollution, especially heavy metal contamination in agricultural lands near industrial zones, which has garnered significant attention due to the serious threats it poses to agricultural ecosystem security and human health [2].

Due to industrial development’s demand for mineral resources, the expanding mining industry poses a significant threat to the surrounding ecological environment. Studies show that the Guangxi Zhuang Autonomous Region is rich in non-ferrous metal resources such as manganese, tin (Sn), palladium (Pd), and zinc (Zn). Mining activities have markedly increased the concentrations of these heavy metal elements in local soils, leading to varying degrees of contamination [3]. Moreover, the use of outdated metal smelting technologies further exacerbates heavy metal pollution. In northwestern Guizhou, long-term reliance on primitive smelting methods has resulted in the release of large quantities of heavy metal including Zn, Pd, and As into the soil, severely affecting local ecosystems and agricultural productivity [4].

Moreover, most industrial parks in China are situated adjacent to agricultural land. During industrial production, substantial amounts of heavy metals enter the soil through atmospheric deposition, wastewater discharge, and solid waste disposal. Since soil microorganisms cannot decompose these metals, they accumulate persistently [5], thereby threatening food security and causing direct and indirect harm to human health. For instance, Zhang et al. [6] reported that crops in an ancient volcanic area of Shandong exhibited the strongest enrichment capacity for As. Similarly, Pu et al. [7] found varying degrees of heavy metal accumulation in soils of a high-geochemical background area in Chongqing, with corn samples showing the most severe exceedance of standard limits. In a lead-zinc mining area of Yunnan, Zhang et al. [8] observed that the exceedance rate for Pb in agricultural products reached 100%. Wu et al. [9] further demonstrated that 35% of crop samples near solid waste sites exceeded heavy metal standards, confirming contamination of adjacent farmland. These findings collectively indicate that heavy metals in soils near mining areas are prone to enrichment in crops, posing long-term health risks. Over decades of development, health risk assessment models have matured and become essential tools for quantitatively assessing risks from human exposure to pollutants. Studies show that heavy metals enter the human body via multiple pathways—such as oral ingestion and inhalation—and their carcinogenic and non-carcinogenic risks vary significantly across populations, including adults and children [10,11,12].

To effectively assess heavy metal pollution in soil, accurate source apportionment is essential. Current methodologies include Geographic Detector, Principal Component Analysis (PCA), Positive Matrix Factorization (PMF), and other multivariate statistical techniques. Combining spatial interpolation with the Geographic Detector can preliminarily identify potential pollution sources, such as historical mining areas. Multivariate methods like Factor Analysis (FA), PCA, and Cluster Analysis (CA) help infer sources by examining correlations between element concentrations [13]. However, like traditional geostatistical methods, these techniques often fail to quantify the specific contribution of individual pollution sources to particular heavy metals and are commonly used to support or validate receptor models. Isotope fingerprinting allows precise traceability but is limited to specific elements [14]. In a study on surface soil from 33 industrial parks in karst landforms, Zhao et al. [15] employed a Monte Carlo model to assess heavy metal contamination, reporting significantly elevated levels of As, Cr, Hg, Ni, Pb, and Zn relative to background values. Based on the potential ecological risk index (RI), half of the industrial parks exhibited moderate risk level. Using the PMF model, the authors quantified the contribution rates of trace element pollution control sources [16].

This study focuses on the Yellow River Bend region, where heavy metal contamination in cultivated soils remains relatively understudied. As a region abundant in land resources and major agricultural production base in China, the quality of its cultivated soil is critical for ensuring agricultural product safety and maintaining regional ecosystem stability. To evaluate the extent of heavy metal pollution resulting from industrial activities such as metal smelting and to assess the associated health risks, this study will implement systematic field sampling and laboratory analysis. This study aims to investigate the current pollution status, identify pollution sources, and perform a comprehensive health risk assessment of heavy metals in the area’s cultivated soils.

## 2. Materials and Methods

### 2.1. Study Area

The study area is located in the Tubo River–Hetao Plain region, bounded by the Yellow River to the south and Mongolia to the north. It serves as a key hub for metallurgical, mechanical, and electrical industries in northern China. The climate is characterized as a semi-arid continental monsoon type, with four distinct seasons—including cold winters, warm summers, and dry spring and autumn periods. The annual average temperature is 34.4 °C; the annual average precipitation is 305 mm, with notable seasonal unevenness in distribution [5]. The region’s pillar industries comprise rare earth mining, steel manufacturing, metallurgy, machinery manufacturing, and the defense related industries.

### 2.2. Sample Collection and Analysis

To ensure representativeness, sampling density was determined according to historical survey data and the spatial distribution of industrial enterprises and cultivated land in the study area. The ArcGIS 10.2 Fishnet tool was used to create a grid of potential sampling points across the region. Grid cells with less than 20% agricultural land coverage were excluded from further consideration. Final sampling points were selected following the “Technical Specifications for Soil Environmental Monitoring” (HJ/T 166-2004) [17]. A double diagonal sampling method was employed, with five sub-sampling points selected within each main sampling location. Sub-samples were collected following standardized procedures, forming a composite sample of approximately 1500 g. Additionally, inorganic parallel samples were collected at each point, resulting in a total sample weight of 2500 g.

**Figure 1:**
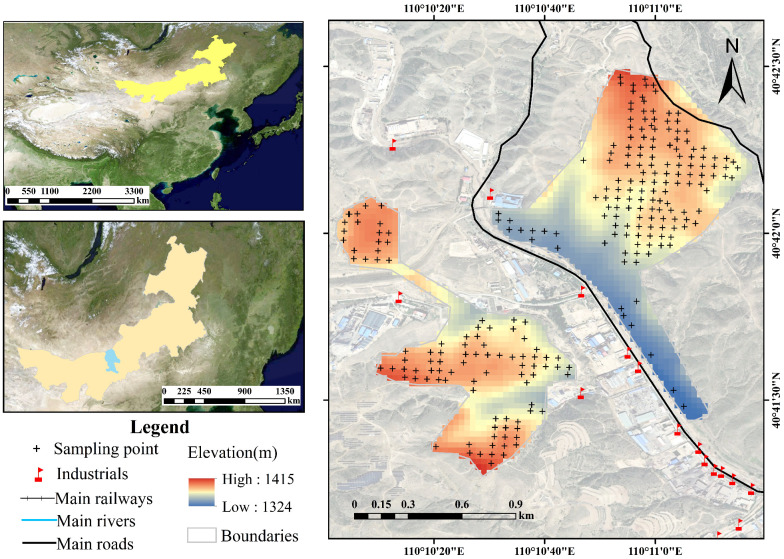
Distribution of sampling locations in the study area.

A sampling grid with a density of 50 m × 50 m was established, and soil samples were collected from cultivated land. A total of 273 surface soil samples (0–20 cm depth) were obtained. The distribution of all samples is shown in Figure 1. To distinguish between natural pedogenic processes and anthropogenic influences on soil element concentrations, four reference sites representing soil environmental background values were selected in nearby areas, using the protocol outlined by the China National Environmental Monitoring Centre [18]. These locations were distant from known pollution sources and shared the same soil parent material as the study area. Sampling was completed in October 2022, covering a total area of approximately 0.58 km^2^.

All collected soil samples were air-dried at ambient temperature, sieved through a 2 mm nylon mesh to remove debris (e.g., brick fragments, limestone nodules, and plant residues), and subsequently ground using a diamond anvil cell until the particles passed through a 0.15 mm nylon sieve. Soil pH was measured using potentiometric titration [19]. For elemental analysis, samples were digested using a microwave-assisted acid mixture of HCl + HNO_3_ + HF + H_2_O_2_. Hg and As concentrations were determined by atomic fluorescence spectrometry (AFS) [20], while Cd, Cr, Pb, Cu, Ni, and Zn concentrations were analyzed using inductively coupled plasma atomic emission spectrometry (ICP-AES) [21].

Laboratory quality assurance and control (QA/QC) were performed following national standard protocols. Certified reference materials GSS-5 and GSS-6 (National Center for Analysis and Testing of Nonferrous Metals and Electronic Materials, Environmental Protection Department’s Standard Sample Research Institute, and Institute of Geophysical and Geochemical Exploration, Chinese Academy of Sciences, Beijing, China) [22]. were used at a frequency of 10% to verify analytical accuracy. In addition, 28 randomly inserted duplicate samples were analyzed to evaluate analytical precision. Recovery rates for metals ranged from 80% to 120%. The method detection limits (MDL) for Hg, Cr, Cu, Pb, Zn, As, Cd, and Ni were 0.002, 4, 1, 10, 1, 0.01, 0.01, and 3 mg·kg^−1^, respectively.

### 2.3. Pollution Assessment

#### 2.3.1. Geoaccumulation Index

The geoaccumulation index (*I_geo_*), also referred to as the Müller index, is employed to assess the degree of heavy metal pollution by comparing current concentrations to their natural background values [23]. It is calculated as shown in Equation (1) and quantifies the extent of anthropogenic enrichment in soils or sediments.

(1) $$I_{geo}=\log_{2}\frac{C_{i}}{{1.5B}_{i}}$$

In this equation, *i* represents the concentration of a certain metal in the sample, while *B_i_* represents the background value of that metal. In this study, the background values from Inner Mongolia region (CNEPA and CNEMC, 1990) [18] (Table S1) were used as references. Based on these values, heavy metal pollution can be classified into seven categories. The specific classification details are shown in Table S2.

#### 2.3.2. Potential Ecological Risk Index

The potential ecological risk index (PERI) is a comprehensive assessment method that incorporates heavy metal concentrations, ecological sensitivity, and toxicological effects. It has been widely applied to evaluate pollution in both lake sediments and soils [24]. The calculations are performed as follows:

(2) $$E_{r}^{i}\;=\;T_{r}^{i}\;\times \;C_{f}^{i}$$

(3) $$E_{r\;}^{i}=\frac{C_{i}}{C_{n}^{i}}$$

where $E_{r}^{i}$ is the ecological risk index of heavy metals, $T_{r}^{i}$ is its toxic response factor, $C_{f}^{i}$ is the contamination factor of heavy metals, and $C_{n}^{i}$ is the soil’s background value in Inner Mongolia. $C_{i}$ is the concentration of the metal *i* in the sample. Toxic response factors for Hg, Cr, Cu, Pb, Zn, As, Cd, and Ni are 40, 2, 5, 5, 1, 10, 30, and 5, respectively [25]. The ecological risk of heavy metals can be classified into five categories (Table S3).

#### 2.3.3. Source Apportionment

Principal Component Analysis (PCA) and Positive Matrix Factorization (PMF) are widely used techniques to analyze heavy metal sources in soil. PCA is a dimensionality reduction technique that transforms multiple correlated variables into a smaller set of linearly uncorrelated principal components through mathematical transformation [26]. It serves as an auxiliary tool for constructing receptor models or validating their results [27]. The PMF model, introduced by Paatero in 2003 [28], is a multivariate factor analysis method that is based on factor analysis principles and source apportionment techniques. This model has been used widely to identify pollutant sources in various environmental media, including soil, water, and air [29,30]. The formula to calculate PMF is presented in Equation (4).

(4) $$E_{nm}=X_{nm}-\sum_{j=1}^{p}{G_{np}F}_{pm}$$

In this equation $X_{nm}$ represents the concentration of the *m*-th chemical component in the *n*-th sample, *p* denotes the number of resolved sources, $G_{np}$ is the source contribution matrix, and $F_{pm}$ is the source composition profile matrix. All elements in matrices $G_{np}$ and $F_{pm}$ are non-negative values, and all parameters in the calculation are dimensionless. The objective function minimum *Q* is calculated as follows:

(5) $$Q\left(E\right)\;=\;\sum_{i=1}^{m}\sum_{j=1}^{n}{\left(E_{ij}/\sigma_{ij}\right)}^{2}$$

When the content is ≤ the corresponding *MDL* (method detection limit):

(6) $$U=\left(5/6\right)\times MDL$$

When the content is > the corresponding *MDL*:

(7) $$U=\sqrt{{\left(s\times c\right)}^{2}+{\left(0.5\times MDL\right)}^{2}}$$

In Equation (7), U represents the measurement uncertainty, *s* denotes the relative standard deviation (RSD), *c* stands for the heavy metal concentration (mg·kg^−1^), and *MDL* indicates the method detection limit (mg·kg^−1^).

#### 2.3.4. Health Risk Assessment

Soil heavy metal pollutants can affect human health through oral ingestion, skin contact, and inhalation. Therefore, Health Risk Assessment (HRA) can use the risk level as an evaluation indicator, combine environmental pollution with human health, and estimate the probability of adverse effects on public health based on the exposure characteristics of the pollutants. The health risk assessment for heavy metals in soil was adapted from the methodology established by the United States Environmental Protection Agency (USEPA, 1999) [31], incorporating the human exposure parameter values specific to the Chinese population, as published by the Ministry of Environmental Protection in 2014 [32], such as the weight (*BW*) of different populations, the skin surface exposure area (*SA*), the Skin Adherence Factor (*AF*), and the Inhalation rate of soil (*InhR*) (to make the assessment more practical). We use correlation to evaluate the amount of heavy metals ingested by adults and children through the following three exposure pathways:

(8) $${ADD}_{ing}\;=\;C_{soil}\;\times \;\frac{IngR\times EF\times ED}{BW\times AT\times 10^{-6}}$$

(9) $${ADD}_{inh}=C_{soil}\;\times \;\frac{InhR\times EF\times ED}{PEF\times BW\times AT}$$

(10) $$\;{ADD}_{der}=C_{soil}\;\times \;\frac{SA\times AF\times ABS\times EF\times ED}{BW\times AT\times 10^{-6}}$$

In this equation, ${ADD}_{ing}$, ${ADD}_{inh}$, and ${ADD}_{der}$, respectively, represent the daily intake of heavy metals through oral ingestion, inhalation, and dermal contact (mg·kg^−1^·d^−1^). *C_soil_* refers to the heavy metal content in the soil (mg·kg^−1^). *IngR* (mg·day^−1^) and *InhR* (m^3^·day^−1^), respectively, represent the frequency of soil intake and inhalation. *EF* (d·a^−1^) and *ED* (a), respectively, represent the exposure frequency and exposure time for different populations. *BW* (kg) represents the weight of different populations. *AT* (d) represents the average exposure time for carcinogenic and non-carcinogenic effects under different exposure pathways for different populations. *PEF* (m^3^·kg^−1^) represents the particle emission factor. *SA* (cm^2^) and *AF* (mg/cm^2^·d), respectively, represent the skin exposure area and skin adhesion factor for different populations. *ABS* represents the skin absorption factor for different populations. The specific values of these parameters can be found in Table S4.

For non-carcinogenic contaminants, *THI* is often used to indicate a risk level for human exposure. For carcinogenic pollutants, *TCR* is often used to indicate the likelihood of inducing cancer when humans are exposed. When contaminants enter through multiple exposure pathways, assuming no synergy or antagonism between them, *HI* and *CR* for all exposure pathways can be calculated as follows [33].

(11) $$THI=\sum {HQ}_{i}=\sum \frac{{ADD}_{i}}{{RfD}_{i}}$$

(12) $${TCR=CR}_{i}=\;{ADD}_{i}\;\times \;{SF}_{i}$$

In this equation, *THI* denotes the hazard index representing non-carcinogenic health risks associated with exposure to eight heavy metal elements. ${HQ}_{i}$ refers to the non-carcinogenic health risk index for individual heavy metal element. *TCR* indicates the total carcinogenic risk for the eight heavy metal elements, while ${CR}_{i}$ represents the carcinogenic health risk index for a specific heavy metals. ${RfD}_{i}$ and ${SF}_{i}$ correspond to the reference dose and the carcinogenic slope factor, respectively, for heavy metals under various exposure pathways. The specific values of these parameters can be found in Table S5 [34].

In the HRA model, when ${HQ}_{i}\;$ or *HI* < 1, it indicates no significant non-carcinogenic risk; when ${HQ}_{i}$ or *HI* > 1, it indicates the existence of non-carcinogenic risk. When ${CR}_{i}$ or *TCR* is less than 10^−6^, it indicates no significant carcinogenic risk; when ${CR}_{i}$ or *TCR* is between 10^−6^ and 10^−4^, it indicates that the carcinogenic risk is within the acceptable range for the human body; when ${CR}_{i}$ or *TCR* > 10^−4^, it indicates the existence of carcinogenic risk.

### 2.4. Statistical Analysis

SPSS 26.0 (SPSS, Chicago, IL, USA) was used for statistical analysis. In ArcGIS10.2 (ESRI, Inc, Redlands, CA, USA), Kriging interpolation was used to study the spatial distribution of heavy metals in soils. Pearson correlation analysis was used to evaluate the relationships between heavy metals. Figures were completed in OriginPro 2022 (OriginLab Corporation, Northampton, MA, USA).

## 3. Results and Discussion

### 3.1. Descriptive Statistics of Heavy Metals in Soils of Agricultural Lands

The concentrations of eight heavy metals (Hg, Cr, Cu, Pb, Zn, As, Cd, and Ni) in the soils are detailed in Table 1. The average soil pH in the study area was 7.7, indicating an overall alkaline condition. Average Hg, Cr, Cu, Pb, Zn, As, Cd, and Ni contents were 0.04 ± 0.05, 48.3 ± 57.6, 54.3 ± 70.2, 45.7 ± 21.9, 70.0 ± 40.7, 22.9 ± 19.1, 0.42 ± 0.70, and 35.7 ± 36.9 mg·kg^−1^, respectively. All heavy metals’ average concentrations exceeded the local background values for Inner Mongolia by 1.32 to 11.21 times. The proportion of sampling sites where concentrations surpassed the background values was 61.5%, 66.3%, 99.6%, 100%, 94.1%, 99.6%, 100%, and 98.90%, respectively. Heavy metal accumulation in these soils varied significantly. Compared with risk intervention values (National Standard for Soil Environmental Quality in China (GB 15618-2018)) [35], the proportion of sites with excessive screening values for eight heavy metals is as follows: As (11.7%), Cd (11.4%), Cu (7.7%), Ni (2.9%), Cr (1.1%), Pb (0.73%), and Zn (0.73%) all exceed the standards; while Hg does not exceed the limit. Compared with the risk intervention values (National Standard for Soil Environmental Quality Standards in China (GB 15618-2018)) [35], the proportion of sites with excessive As was 0.37%; the proportion of sites with excessive Cd was 1.1%; and Hg, Cr, and Pb did not exceed the risk intervention values. Overall, the degree of soil pollution was relatively mild, but they may pose threats to agricultural production and public health. High concentrations of heavy metals in soils (and high coefficients of variation (CV)) suggest anthropogenic disturbance of the soil environment [36,37]. There are four levels of variation [38]: low, moderate, high, and extreme (Tables S6 and S7). CVs for Hg, Cr, Cu, Cd, and Ni concentrations were very high (107%, 119%, 129%, 166%, and 103%, respectively), with this extremely uneven distribution of metals indicating exogenous input [39]; CVs for Zn ranged from 50% to 100%. The high variation in heavy metal contents and their large spatial dispersion indicates that these soils are greatly affected by anthropogenic disturbance [40]. In general, As, Cd, and Cu were polluted in the area.

### 3.2. Spatial Distribution of Heavy Metals in Soils

**Figure 2:**
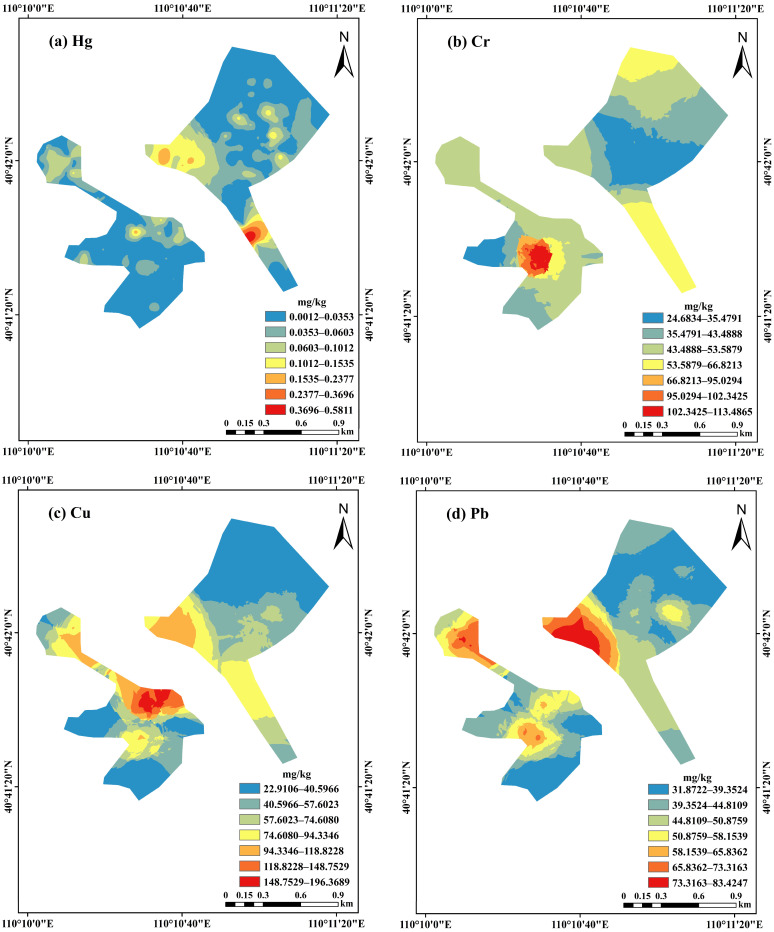

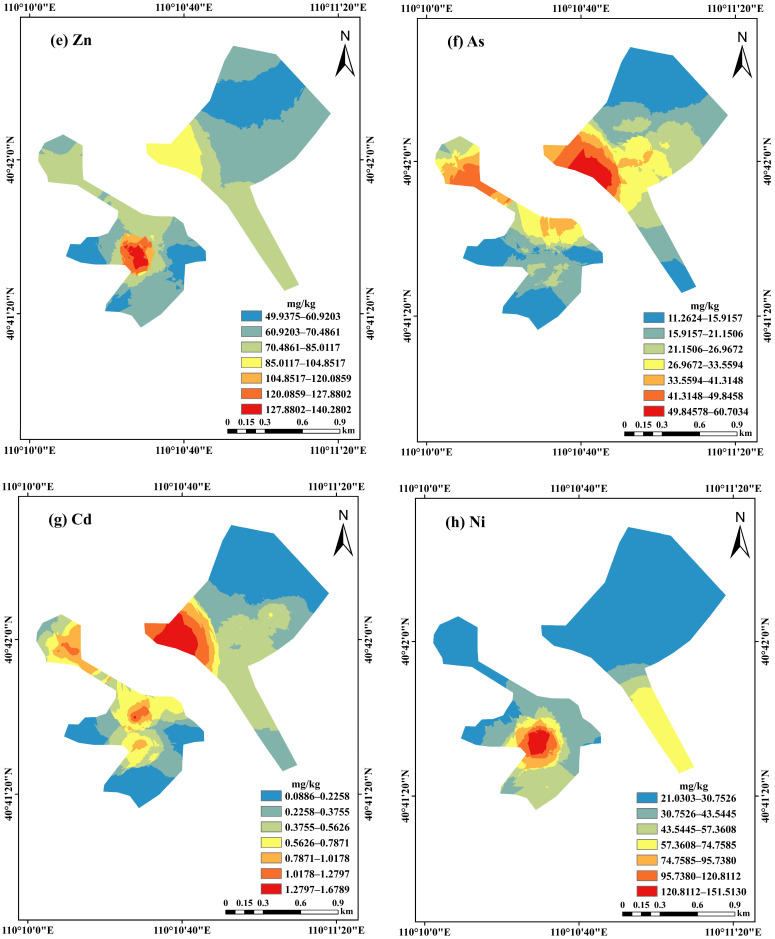
Spatial distributions of heavy metal concentrations.

Interpolation was performed to interpret the distributions throughout the study area. Based on sampling locations, the surveyed region was divided into eastern and western parts. To fit the variogram model, ordinary Kriging interpolation was performed for heavy metal contents in soils. Interpolation results are shown in Figure 2. Heavy metal distributions throughout the study area are generally similar; in the western part they occur mainly in the upper middle survey region, and in the eastern part, mainly in the lower middle survey region. The high-value distribution of Hg is relatively concentrated in the eastern lower part of the study area. Due to its high mobility, Hg tends to accumulate in this region, resulting in elevated concentrations. The contents of Cu, Pb, As, and Cd are similarly distributed in eastern (higher in the middle and lower region than that in the upper region) and western (higher in the middle and upper region than in the lower region) areas of the survey region. According to the Pearson correlation analysis (Figure S1), at the 0.01 significance level, Cu-Cd (r = 0.75), Pb-Cd (r = 0.83), Pb-As (r = 0.77), and As-Cd (r = 0.78) exhibit highly significant positive correlations. This suggests that these four heavy metals may originate from the same or similar sources [41,42,43]. The distributions of Cr, Zn, and Ni were similar, with high contents concentrated in the central western area, and decreasing from the center of the survey region. According to the Pearson correlation analysis (Figure S1), at the 0.01 significance level, Zn-Cr (r = 0.71) and Zn-Ni (r = 0.63) exhibit highly significant positive correlations. This suggests that Cr and Zn may originate from the same or similar sources. Additionally, high Cu, Pb, As, and Cd contents are most widely distributed. Many mineral products arise from processing plants around the study area, mostly engaged in Pb-Zn and Cu mining and smelting; wastes from the production process are important sources of Cu, Pb, As, and Cd [44]. Additionally, the high-value distributions of Cu, Pb, As, and Cd are most widely distributed. Field investigations in the study area identified several ferroalloy smelting enterprises located in the southeastern region. Heavy metal-laden smoke and dust generated during the smelting process (e.g., Cr, Pb, Cd, As) are released into the atmosphere, where they disperse and eventually deposit onto the soil surface [45]. The prevailing wind direction in the study area is from the southeast, and elevated concentrations of heavy metals are predominantly found in the upper and middle parts of the study area.

Overall, medium to high ecological risk is predominantly concentrated in the upper-middle portion of the western region and the central-left portion of the eastern region. When comparing the spatial distribution of HMs, it is evident that Cr, Pb, As, and Cd exhibit distributions similar to that of the overall ecological risk distribution. Based on the calculated *I_geo_* for heavy metals, Cd, Cu, and As are identified as the most significant pollutants, exhibiting severe contamination levels and posing a high potential ecological risk.

### 3.3. Combining PCA and PMF Models to Jointly Identify the Sources of Heavy Metals

The results of principal component analysis are shown in Table 2. Three principal components with a cumulative contribution of 81.39% were extracted, which can reflect the information of the raw data comprehensively [27]; the KMO value for the model was 0.768, and the Bartlett’s test was <0.01. The first principal component (PC1) accounts for 48.22% of the total variance, with Cd, As, Cu, and Pb identified as the dominant indicator elements. The second principal component (PC2) explains 22.71% of the variance, with Ni, Zn, and Cr as the primary indicators. The third principal component (PC3) contributes 10.46% to the total variance, with Hg as the sole indicator element.

**Figure 3:**
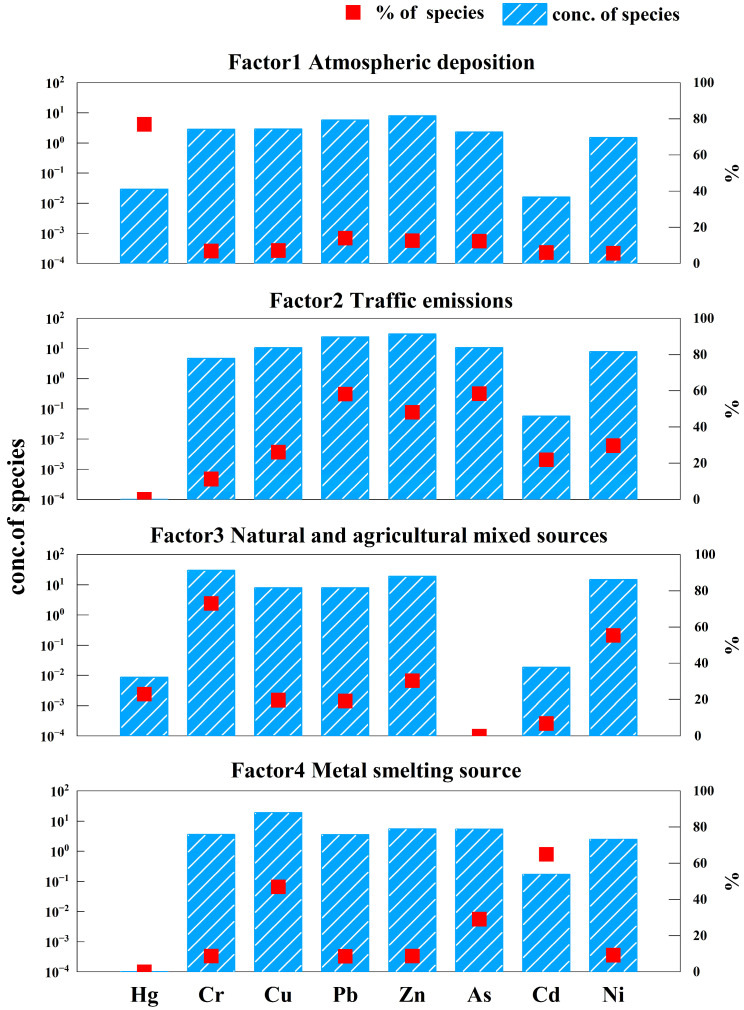
Source apportionment and factor contribution of heavy metals in soils.

PCA is a new variable established based on the close relationship between the original data [46]. Because the accumulation of heavy metals for a PC may have similar sources, the eight heavy metals that we report from these soils could be derived from at least three sources. To explore this possibility, we used a PMF model to analyze the source of metals and to determine the contribution of each. A total of 273 groups of heavy metal concentrations and uncertainties were imported into the PMF model. Because the number of samples exceeded 100, the signal-to-noise ratio was “strong,” and for five metals (excluding Cu, Cd, and Ni), the residuals of the other five heavy metals were normally distributed (fitting coefficients > 0.70). The contribution factor of the PMF model increased from 3 to 5 in turn. By comparing the ratio of *Q* (robust) and *Q* (true), the *Q* value of the four contribution factors is set to be closest to 1, so we believe that the four contribution factors best explain heavy metal concentrations (Figure 3).

The contribution of the four factors from greatest to least was F2 > F3 > F4 > F1, with that of F1 being 10.10%. Hg is a characteristic metal in factor 1. The contribution rate of F2 was 38.19%, and As, Pb, and Zn are characteristic heavy metals for this factor. The contribution rate of F3 is 34.55%, and Cr, Ni, and Zn are characteristic metals in this factor. The contribution rate of F4 was 17.16%, and Cd, Cu, and As are characteristic heavy metals in this factor. The contribution of F1 corresponds to PC3, that of F3 corresponds to PC2, and those of F2 and F4 are the further analysis of PC1.

The contribution rates of F1 and Hg were 10.10% and 80.4%, respectively. The CV of Hg was high (107%), indicating that it may be affected by human or natural factors. Hg in soil is mainly sourced from industrial activities such as fossil fuel combustion and non-ferrous metal smelting via atmospheric deposition [47,48]. High concentrations of Hg occur mainly in the southeast of the survey area near a large industrial park with more than 20 smelting enterprises, mostly producing ferrosilicon, ferronickel, and crude copper. Smelting consumes considerable coal and produces considerable flue gas that contains heavy metals such as Hg. The prevailing winds in the study area are southeasterlies, and Hg is highly mobile [49], entering the soil via atmospheric deposition. Therefore, we infer that F1 represents atmospheric deposition as a source.

Among the four identified factors, F2 exhibited the highest contribution rate (38.19%). The heavy metals As (58.41%), Pb (58.16%), and Zn (48.16%) were predominantly loaded on a single factor, indicating a distinct pollution source. Traffic exhaust emissions are important sources of Pb in agricultural soils, with vehicle exhaust contributing about two-thirds of global Pb emissions [50,51]. Cu may be derived from vehicle brakes and Zn from tire wear [52]. We infer that F2 represents emissions from traffic.

Factor 3 accounted for 34.55% of the total contribution, ranking second among the identified sources. It was predominantly characterized by Cr (73.01%) and Ni (55.40%). Although the average Cr content (47.70 mg·kg^−1^) was lower than the local background value (56.63 mg·kg^−1^)—indicating an absence of significant Cr pollution—spatially, elevated concentrations of both Cr and Ni were primarily observed in cultivated areas. In addition, F3 included considerable contributions from As (29.19%) and Cu (46.86%), which are commonly associated with agricultural activities such as the application of pesticides, chemical fertilizers, and commercial organic fertilizers. The long-term and extensive use of these agrochemicals likely leads to the accumulation of these metals in surface soils. Therefore, F3 is interpreted as a mixed source derived from both natural geological background and agricultural practices.

The contribution rate of F4 was 17.16%, with the contributions of its characteristic elements Cd (64.97%), Cu (46.96%), and As (29.19%). Cd, Cu, and As are typical industrial metal smelting pollutants. By comparing data for heavy metals in soils and surface water, the Cd and Zn contents in our study area are very high, although Cd and Zn contents in surface water basically meet farmland irrigation water quality requirements. High concentrations of these three metals occur mainly in the middle and upper western district and the left and central eastern district. The study area is rich in mineral resources, and metal smelting enterprises, alloy manufacturers, and manufacturers of graphite and carbon products form a complete enterprise chain. Because metal smelting and industrial combustion activities produce Cd pollution [53], we identify the source of metals in F4 to be metal smelting.

### 3.4. Risk Assessment

#### 3.4.1. Assessment of Soil Heavy Metal Pollution and Potential Ecological Risks

**Figure 4:**
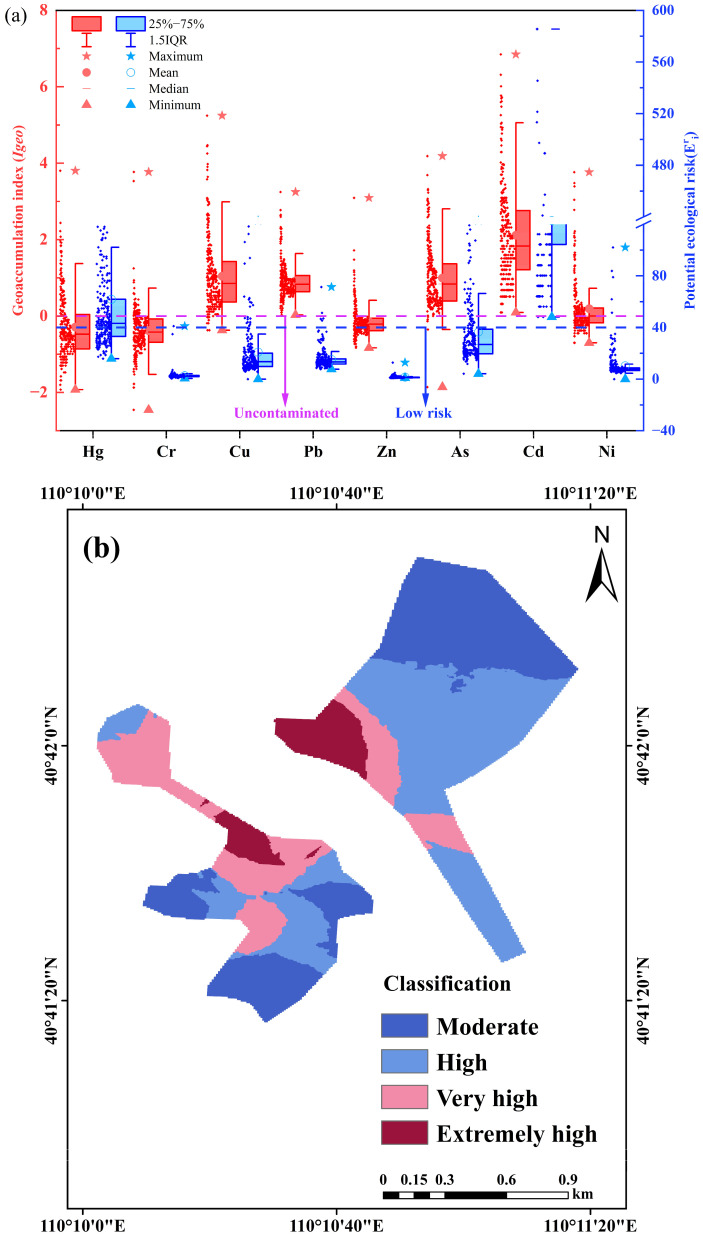
Risk assessment results of heavy metals in soil (**a**) the results of geoaccumulation index (*I_geo_*) and the potential ecological risk index (*RI*) (**b**) the spatial distribution of potential ecological risk index.

Figure 4 presents the evaluation results of the geoaccumulation index and the potential ecological risk index of heavy metals, along with the spatial distribution of the potential ecological risks. According to the soil geoaccumulation index classification, over 75% of sampling sites for Cu, Pb, and As exhibited light to moderate pollution, whereas more than 75% of sites for Cd indicate moderate to high contamination levels. The potential ecological risk index results demonstrated that over 75% of sites for Cr, Cu, Pb, Zn, and Ni presented low potential ecological risk, whereas over 75% of the sites for Cd were classified as high potential ecological risk. Combining this with the spatial distribution of the potential ecological risk, areas with high ecological risk are primarily concentrated in the upper and middle regions on the western side of the study area, while the potential ecological risk index gradually decreases from left to right on the eastern side. The overall spatial pattern of the RI closely resembles the spatial distribution of Pb, As, and Cd concentrations. Therefore, we should be focused on the contamination of Cd, As and Pb.

#### 3.4.2. Integrated Metal-Source-Risk Analysis

Total non-carcinogenic risk indices for children (0.12) and adults (0.065) were <1, indicating an acceptable risk to both. However, the total non-carcinogenic risk index for children was higher than that for adults (Table S6), possibly because of differences in children’s behavior and environmental exposure routes (contact) [49]. The cancer risk index for children (6.70 × 10^−5^) and adults (5.67 × 10^−5^) was <1 × 10^−4^ (a limit), indicating that carcinogenic risks were within acceptable limits. For both non-carcinogenic and carcinogenic risks, oral ingestion was identified as the primary exposure pathway.

**Figure 5:**
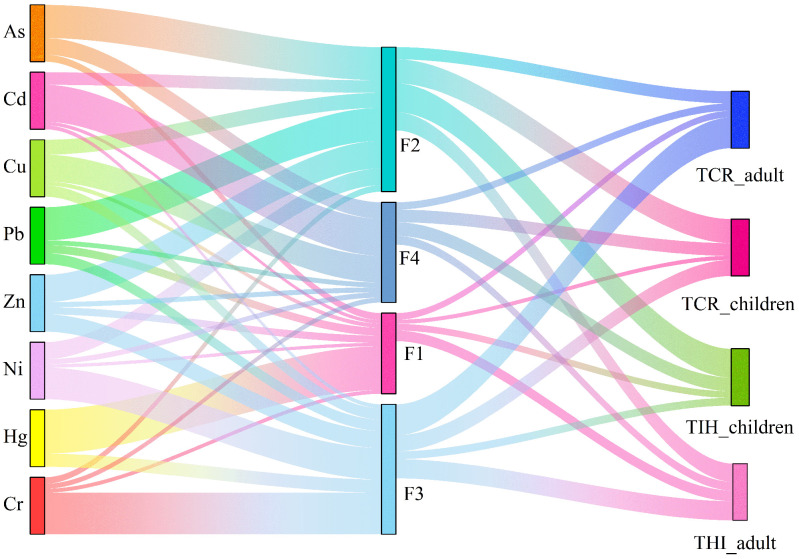
Sankey diagram of the relationship among heavy metals, sources, and health risks (The width is proportional to the contribution level).

Based on HRA results, a Sankey diagram was constructed to illustrate the relationships between heavy metals, pollution sources, and health risks (Figure 5). For non-carcinogenic risk, the contribution order of the four sources was F2 > F3 > F4 > F1; the contribution rates of health risks to adults and children from various sources are as follows: atmospheric deposition accounts for 18.71% (11.67%), traffic emissions contribute 32.12% (50.59%), natural and agricultural mixed sources account for 33.76% (13.69%), and metal smelting sources contribute 15.41% (24.05%). For carcinogenic risk, the source contribution order was F3 > F2 > F4 > F1; the contribution rate of health risk to adults (children) was 11.79% (7.16%), 21.39% (42.37%), 53.95% (28.24%), and 12.86% (22.23%) for atmospheric deposition sources, traffic emissions sources, and natural and agricultural mixed sources, respectively.

Among the carcinogenic risks, traffic emissions and natural and agricultural mixed sources contributed most significantly to both adults and children. From the perspective of pollutants, As, Pb, Zn, Cr, and Ni warrant priority attention and control due to their substantial contributions to human health risks [54]. According to relevant studies, As and Cr have high bioavailability and toxicity [49]. As is classified as a Group 1 carcinogen by the World Health Organization, is a priority pollutant in many countries, and is associated with lung cancer, skin cancer, and liver cancer [55]. Its primary exposure pathways include direct ingestion, dermal contact, and inhalation [56]. Cr exposure occurs mainly through oral intake, with excessive levels causing dermatological and respiratory damage [57]. Combined with Section 3.4.1, heavy metal Cd shows the characteristics of high ecological risk and low carcinogenic contribution. While metal smelting sources rank third in carcinogenic contribution, their overall impact on human health remains relatively modest. Nevertheless, Cd is highly toxic, and related studies have shown that Cd can damage local ecosystems through contaminated soil and drinking water, causing serious harm to humans and animals [58]. Overall, agricultural and transportation activities represent the primary sources of health risks in the region. Although metal smelting’s direct impact on human health is comparatively smaller, Cd pollution requires ongoing attention. Therefore, to protect human health from carcinogenic risks, local transportation management and the use of fertilizers and pesticides in agricultural activities should be strengthened. Additionally, for Cd pollution, it is recommended to conduct sampling of local agricultural products for further analysis of heavy metal pollution.

## 4. Conclusions

Overall, cultivated soils in the study area exhibit significant heavy metal contamination. Geoaccumulation and potential ecological risk indices reveal severe pollution from Cd, Cu, As, and Pb, with Cd posing the highest ecological risk. Four primary sources contribute to this contamination: atmospheric deposition, traffic emissions, mixed natural-agricultural sources, and metal smelting. Traffic emissions and mixed natural–agricultural sources collectively account for >70% of total heavy metal inputs. Analysis using a Sankey diagram reveals that these two dominant sources also represent the greatest threats to human health. Although metal smelting ranks third in contribution, its health implications remain substantial and warrant attention. To better assess the risks of contamination transfer to the food chain and human health, additional sampling of local crops is recommended.

In this study, the soil samples were collected only from the surface layer (0–20 cm), which could not fully reflect the vertical migration of heavy metals and their accumulation in the deeper soil layers. Therefore, it might underestimate the potential pollution risk of heavy metals to groundwater and the long-term impact on the ecosystem. Moreover, this study did not conduct simultaneous sampling and analysis of local agricultural products and is thus unable to assess the migration and accumulation effect of heavy metals from the soil to crops and their contribution to dietary exposure risks. This limitation restricts a more comprehensive and accurate assessment of the health risks to the population. Future research should combine profile soil sampling and agricultural product monitoring to improve the systematic understanding of the migration and transformation patterns of heavy metals and the transmission paths of health risks.

## Figures and Tables

**Table 1:** Descriptive statistics of heavy metal concentrations in the agricultural soil (*n* = 273).

Parameter	pH	*w*/mg·kg^−1^							
		Hg	Cr	Cu	Pb	Zn	As	Cd	Ni
Min	6.5	0.01	10	15	23	41	2.6	0.06	16
Max	8.9	0.58	748	734	214	622	172	6.5	353
Mean	7.7	0.04	48.3	54.3	45.7	70.0	22.9	0.42	35.7
SD	0.58	0.05	57.6	70.2	21.9	40.7	19.1	0.7	36.9
CV/%	8	107	119	129	48	58	83	166	103
>background value/% ^a^	-	61.5	66.3	99.6	100	94.1	99.6	100	98.9
>risk screening value/% ^b^	-	0	1.1	7.7	0.73	0.73	11.7	11.4	2.9
>intervention value/% ^b^	-	0	0	-	0	-	0.37	1.1	-

^a^ Soil background of Mongolia. ^b^ National Standard for Soil Environmental Quality in China (GB 15618-2018) [35].

**Table 2:** Factor load of heavy metal elements in soil. (after 4 times of rotation convergence).

Elements	PC		
	1	2	3
Cd	0.917	0.155	0.143
Hg	0.233	0.043	0.959
As	0.877	0.007	0.178
Pb	0.871	0.242	0.177
Cr	−0.125	0.877	0.075
Cu	0.830	0.099	0.012
Ni	0.231	0.754	−0.078
Zn	0.301	0.884	0.102
Characteristic value	3.858	1.817	0.837
Variance contribution rate (%)	48.23	22.71	10.46
Cumulative variance contribution rate (%)	48.23	70.94	81.39

## Data Availability

The data presented in this study are available on request from the corresponding author.

## Supplementary Materials

The following supporting information can be downloaded at: https://www.mdpi.com/article/10.3390/toxics13100834/s1, Table S1: The risk screening values for soil contamination and background values of agricultural lands. Table S2: Classification criteria of the geo-accumulation pollution index. Table S3: Classification criteria of the Potential ecological risk index. Table S4: Reference values of parameters in human health risk assessment. Table S5: Values of reference dose (mg·kg^−1^·day^−1^) and slope factor (mg·kg^−1^·day^−1^) for heavy metals [34]. Table S6: Variation coefficient degree table. Table S7: Statistical results of health risk caused by heavy metals in soil. Figure S1: The correlation of heavy metals in the soils of the study area.
