# Supplementary material for: Heavy Metal Pollution and Health-Ecological Risk Assessment in Agricultural Soils: A Case Study from the Yellow River Bend Industrial Parks
Source: Toxics. 2025 Sep 30;13(10):834. doi: 10.3390/toxics13100834 (PMC12567825; doi:10.3390/toxics13100834)
Supplement: Supplementary file 1 [file toxics-13-00834-s001.zip › toxics-3806796-supplementary.pdf]

Supplementary material

Table S1 The risk screening values for soil contamination and background values of agricultural lands

Heavy metal	risk screening value for soil contamination				background value
	pH≤5.5	5.5<pH≤6.5	6.5<pH<7.5	pH>7.5	
Hg	1.3	1.8	2.4	3.4	0.03
Cr	150	150	200	250	36.5
Cu	50	50	100	100	12.9
Pb	70	90	120	170	15
Zn	200	200	250	300	48.6
As	40	40	30	25	6.3
Cd	0.3	0.3	0.3	0.6	0.04
Ni	60	70	100	190	17.3

Table S2 Classification criteria of the geo-accumulation pollution index

I_{geo}	Class	Degree of contamination
$I_{geo} \leq 0$	0	uncontaminated
$0 < Igeo \leq 1$	1	uncontaminated to moderately contaminated
$1 < Igeo \leq 2$	2	moderately contaminated
$2 < Igeo \leq 3$	3	moderately to heavily contaminated
$3 < Igeo \leq 4$	4	heavily contaminated
$4 < Igeo \leq 5$	5	heavily to extremely contaminated
$5 < Igeo \leq 6$	6	extremely contaminated

Table S3 Classification criteria of the Potential ecological risk index

potential ecological risk, E_r^i	potential ecological risk index, RI	classification of potential eco-risk
$E_r^i < 40$	$RI < 150$	Low
$40 \le E_r^i < 80$	$150 \le RI < 300$	Moderate
$80 \le E_r^i < 160$	$300 \le RI < 600$	High
$160 \le E_r^i < 320$	$600 \le RI < 1200$	Very high
$E_r^i \ge 320$	$RI \ge 1200$	Extremely high

Table S4 Reference values of parameters in human health risk assessment

Symbol	Parameter	Value	Reference
C_{soil}	Mean	mg/kg	This study
IngR	Ingestion rate	100 mg/day for adults;200 mg/day for children	USEPA 2002
ED	Exposure duration	24 years for adults;6 years for children	USEPA 2002
EF	Exposure frequency	350 days for adults and children	USEPA 2002
$AT_{non-caarcinogenic}$	Average time	365×ED for adults and children	USEPA 2002
BW	Body weight	56.8 kg for adults;15.9 kg for children	MEP 2014
SA	Exposed skin surface area	5075 cm^2 for adults;2447 cm^2 for children	MEP 2014
AF	Skin adherence factor	0.07 $mg/cm^2 \cdot d$ for adults;0.2 $mg/cm^2 \cdot d$ for children	MEP 2014
ABS	Dermal absorption factor	0.001 for all elements except As is 0.03	USEPA 2011
InhR	Inhalation rate of soil	15 m^3/day for adults;7.5 m^3/day for children	MEP 2014
PEF	Particle emission factor	1.36*109 m^3/kg^{-1} for adults and children	USEPA 2002
$AT_{caarcinogenic}$	Average time	365*70 for adults and children	Abdelhafez and Li 2015

Table S5 Values of reference dose (mg·kg^{-1}·day^{-1}) and slope factor (mg·kg^{-1}·day^{-1}) for heavy metals

Element	RfDing	RfDder	RfDinh	SFing	SFder	SFinh	RfC
Hg	3.00E-04	2.10E-05	8.57E-05	-	-	-	3.00E-04
Cr	3.00E-03	6.00E-05	2.86E-05	5.00E-01	-	4.20E+01	1.00E-04
Cu	4.00E-02	1.20E-02	-	-	-	-	-
Pb	-	-	-	-	-	-	-
Zn	3.00E-01	6.00E-02	-	-	-	-	-
As	3.00E-04	1.23E-04	4.29E-06	1.50E+00	3.66E+00	1.51E+01	1.50E-05
Cd	1.00E-03	1.00E-05	1.00E-05	2.00E+01	3.80E-01	6.30E+00	1.00E-05
Ni	2.00E-02	5.40E-03	9.00E-05	-	-	8.40E-01	9.00E-05

Reference

Lingfeng Zhou, Xiaoli Zhao, Yaobin Meng, Yang Fei, Miaomiao Teng, Fanhao Song, Fengchang Wu,Identification priority source of soil heavy metals pollution based on source-specific ecological and human health risk analysis in a typical smelting and mining region of South China,Ecotoxicology and Environmental Safety,Volume 242, 2022,113864,ISSN 0147-6513, https://doi.org/10.1016/j.ecoenv.2022.113864.

Table S6 Variation coefficient degree table

CV%	Variability Level
CV≤20%	Low variability
20%<CV≤50%	Moderate variability
50%<CV≤100%	High variability
CV>100%	Extreme variability

Table S7 Statistical results of health risk caused by heavy metals in soil.

		Non-carcinogenic risk				Carcinogenic risk			
	element	HQ_{ing}	HQ_{der}	HQ_{inh}	HQ	CR_{ing}	CR_{der}	CR_{inh}	CR
Children	Hg	1.48E-04	5.17E-06	1.43E-14	1.53E-04	-	-	-	-
	Cr	1.66E-02	2.04E-03	4.81E-11	1.87E-02	2.50E-05	-	5.78E-14	2.50E-05
	Cu	1.40E-03	1.14E-05	-	1.41E-03	-	-	-	-
	Pb	-	-	-	-	-	-	-	-
	Zn	2.41E-04	2.95E-06	-	2.44E-04	-	-	-	-
	As	7.90E-02	1.41E-02	0.001398	9.32E-02	3.56E-05	6.37E-06	9.06E-08	4.20E-05
	Cd	4.33E-04	1.06E-04	1.20E-12	5.39E-04	-	-	7.53E-17	7.53E-17
	Ni	1.84E-03	1.66E-05	1.13E-11	1.85E-03	-	-	8.51E-16	8.51E-16
Adults	Hg	1.40E-04	7.10E-06	5.40E-14	1.47E-04	-	-	-	-
	Cr	2.91E-02	5.17E-03	3.36E-10	3.42E-02	4.36E-05	-	4.04E-13	4.36E-05
	Cu	4.02E-04	4.76E-06	-	4.07E-04	-	-	-	-
	Pb	-	-	-	-	-	-	-	-
	Zn	1.91E-04	3.40E-06	-	1.95E-04	-	-	-	-
	As	2.29E-02	5.96E-03	0.001623469	2.89E-02	1.03E-05	2.68E-06	1.05E-07	1.31E-05
	Cd	7.21E-05	2.56E-05	7.95E-13	9.77E-05	-	-	5.01E-17	5.01E-17
	Ni	1.05E-03	1.38E-05	2.57E-11	1.06E-03	-	-	1.95E-15	1.95E-15

Figure. S1 The correlation of heavy metals in the soils of the study area

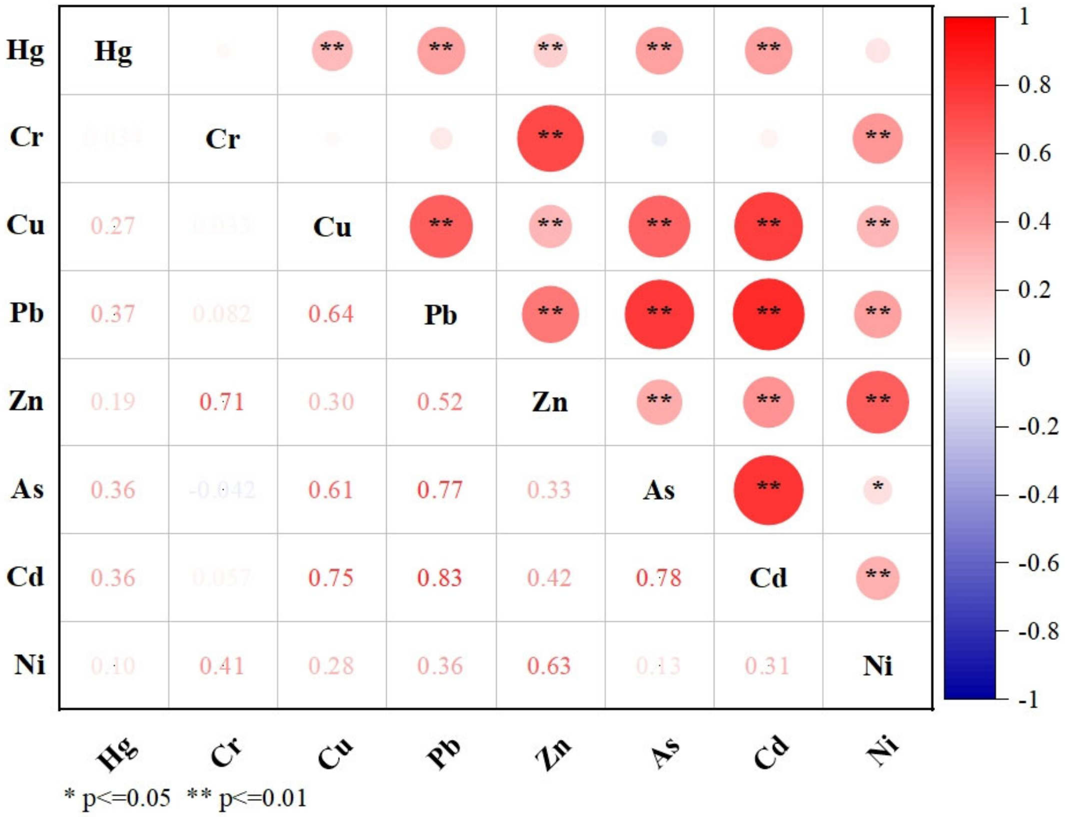

Standard substance

GSS-5 and GSS-6 were produced by the National Center for Analysis and Testing of Nonferrous Metals and Electronic Materials, the Environmental Protection Department's Standard Sample Research Institute, and the Institute of Geophysical and Geochemical Exploration (IGGE) of the Chinese Academy of Sciences.

Reference

Lei Chai, Yuhong Wang, Xin Wang, Liang Ma, Zhenxiang Cheng, Limin Su,
Pollution characteristics, spatial distributions, and source apportionment of heavy metals in cultivated soil in Lanzhou, China, Ecological Indicators, 2021,125, https://doi.org/10.1016/j.ecolind.2021.107507.
